# Tanapox, South Africa, 2022

**DOI:** 10.3201/eid2906.230326

**Published:** 2023-06

**Authors:** Monica Birkhead, Wayne Grayson, Antoinette Grobbelaar, Veerle Msimang, Naazneen Moolla, Angela Mathee, Lucille Blumberg, Terry Marshall, Daniel Morobadi, Mirjana Popara, Jacqueline Weyer

**Affiliations:** National Institute for Communicable Diseases, Johannesburg, South Africa (M. Birkhead, A. Grobbelaar, V. Msimang, N. Moolla, L. Blumberg, J. Weyer);; Ampath Laboratories, Centurion, South Africa (W. Grayson, T. Marshall, D. Morobadi);; University of the Witwatersrand, Johannesburg (W. Grayson, J. Weyer);; South African Medical Research Council, Cape Town, South Africa (A. Mathee);; University of Johannesburg, Johannesburg (A. Mathee);; University of Pretoria, Pretoria, South Africa (N. Moolla, L. Blumberg, J. Weyer);; Right to Care, Johannesburg (L. Blumberg);; University of the Free State, Bloemfontein, South Africa (D. Morobadi);; Mediclinic, Sandton, South Africa (M. Popara)

**Keywords:** Yatapoxvirus, Tanapoxvirus, South Africa, zoonotic virus, zoonoses, viruses, light microscopy, electron microscopy, PCR, DNA sequencing, vector-borne infections

## Abstract

Tanapox is a rarely diagnosed zoonosis known to be endemic to equatorial Africa. All previously reported human cases were acquired within 10° north or south of the Equator, most recently 19 years ago. We describe a human case of tanapox in South Africa (24° south of the Equator). Expanded surveillance for this pathogen is warranted.

Tanapox is a rarely diagnosed zoonosis endemic to equatorial Africa. Only 4 exported cases from Africa involving either human or nonhuman primates have been reported (Table). Initial human outbreaks, in 1957 and 1962, were recorded from the Tana River Valley of Kenya, and the etiologic agent was subsequently isolated and described as Tanapox virus (TANV) (genus *Yatapoxvirus*, family *Poxviridae*) in 1965 (*1*). Subsequently, the occurrence of tanapox in research laboratory primates imported into the United States (*2*,*3*) led to a serologic survey of 12 nonhuman primate species from Kenya, Ethiopia, Cameroon, Côte d’Ivoire, Liberia, and Senegal; seropositivity was detected in all species surveyed in those countries (*8*). Therefore, nonhuman primates across equatorial Africa were surmised to be the natural reservoirs of TANV and humans incidental hosts (*3*,*8*). On the basis of the overlap between human tanapox cases and the geographic ranges of selected nonhuman primates, an ecologic niche model predicted that tanapox could be found from Somalia to Senegal, with the most southerly range above the Tropic of Capricorn (*9*).

**Table:** History of recorded tanapox cases in humans and nonhuman primates, 1957–2004

Year	Location of exposure	Epidemiologic description	Reference
1957	Ngau, Kenya (Tana River Valley)	Several Wapakomo school children diagnosed with tanapox	(*1*)
1962	Between Garissa and Garsen, Kenya (Tana River Valley)	About 50 case-patients from the Wapakomo tribe	(*1*)
1965–1966*	Holding facilities of primate supplier, USA	Infected macaques from the same supplier, distributed to 3 primate research centers in Oregon, California, and Texas, USA	(*2*–*4*)
1966–1968†	Laboratory-acquired	Several laboratory workers in Oregon and California became infected after handling of laboratory macaques	(*2*–*4*)
1971†	Laboratory-acquired	Human volunteer was inoculated with tanapox virus, and clinical progression of the disease was monitored and recorded	(*1*)
1979–1983	Mongala, Democratic Republic of Congo (then Zaire)	A total of 357 cases reported, of which 264 were confirmed by laboratory testing	(*3*)
1999	Bagamoyo, Tanzania	Traveler from Germany diagnosed with tanapox upon return from Tanzania	(*5*)
2002†	Sierra Leone	Person from Sierra Leone admitted to hospital in New York, USA, 2 weeks after arrival from Sierra Leone	(*6*)
2004	Republic of Congo	Volunteer working with chimpanzees has onset of tanapox; only diagnosed after return to USA	(*7*)

*Initially identified as Yaba-like disease virus; subsequent research indicated homology with *Tanapoxvirus.*†Date of report (date of actual case not published).

The epidemiology and natural ecology of tanapox is largely unknown, but previous reports indicate that all infected humans are equally affected, regardless of age group and sex. Serologic surveys conducted in Tana River communities indicated 16.3% prevalence in 1971 and 9.2% in 1976 (*10*). Human-to-human transmission is rare, and although transmission from nonhuman primates to humans by contact or inoculation has been noted under laboratory conditions, natural human infections are more likely to be acquired by mechanical transmission from contaminated mouthparts of hematophagous arthropods (*2**–**4*). This vector theory arose because of the synchronicity between tanapox outbreaks and the increased arthropod activity associated with seasonal high temperatures, high rainfall, and flooding in the riparian areas in which surveillance was done (*1*,*3*,*9*). The similarities in the distribution and incidence of TANV and West Nile virus antibodies in serum samples collected in the Tana River Valley in 1971 led to the suggestion that both viruses are transmitted in the same way (i.e., by a culicine mosquito, probably a species of *Mansonia*) (*1*,*3*).

In humans, tanapox typically manifests with 1 or 2 characteristic, nodular skin lesions that are large, raised, umbilicated, and painful and generally ulcerate without becoming pustular (in contrast to lesions observed in most other poxvirus infections) (*1*,*3*,*7*). The lesions may be associated with localized lymphadenopathy, and their gradual development is preceded by a mild, short-lived febrile illness, with possible pruritus and myalgia leading to prostration and headaches (*1*–*3*,*5*,*6*). Histologically, lesions are restricted to the epithelial layers, with cells containing eosinophilic cytoplasmic inclusions and vacuolated nuclei (*1*,*2*,*7*). TANV virions are poxlike but cannot be distinguished microscopically from the other species in the genus (e.g., Yaba monkey tumor virus) or from *Orthopoxvirus* virions (*3*,*7*). Clinical differential diagnoses have included cutaneous anthrax, other poxvirus infections, sporotrichosis, *Mycobacterium marinum* infection, spotted fever group rickettsial infections, tropical ulcers, insect bites, and scabies (*3*,*7*,*9*). The disease is self-limiting, with no recorded fatalities (*3*). We describe a case of tanapox in South Africa, 19 years after the last published report of human tanapox (*7*).

## The Study

**Figure 1:**
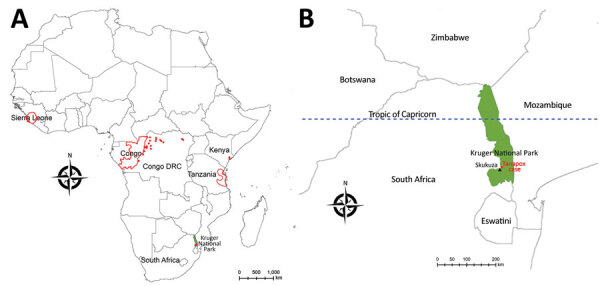
Geographic distribution of recorded human cases of tanapox. A) Locations of previous tanapox cases reported in the literature. Red dots indicate cases acquired locally; red outlines indicate regions of countries visited by travelers to Africa. B) Location of the case acquired in Kruger National Park, South Africa, 2022. Green shading shows the park’s location; black triangle indicates town of Skukuza.

We obtained written consent from the patient in this study and received ethics clearance from the Faculty of Health Sciences of University of the Witwatersrand, Johannesburg (approval no. M210752). During February 2–6, 2022, a 61-year old woman served as a volunteer in Kruger National Park (KNP), South Africa. She stayed in a tented bush camp along the banks of the Sand River, ≈20 km from the town of Skukuza (24°59′43′′ S, 31°35′34′′E) (Figure 1; Appendix Figure 1). The woman noted large numbers of arthropods (e.g., spiders, insects, and ticks) around the camp site and reported being bitten on several occasions on various body parts (especially on her hands, shoulders, arms, and back). Because of heavy rains, trails were overgrown, so bushwalks resulted in scratches on her arms, and many ticks were found on her clothing. During February 7–9, she continued her visit to KNP as a guest in air-conditioned accommodation and had no further direct contact with vegetation. She reported no direct contact with primates, although vervet monkeys (*Chlorocebus pygerythrus*) are seen near the camps. 

Two days after her return to urban Johannesburg, she experienced pruritus at the base of her thumb on the dorsal side of her right hand and noticed a pale blister forming there, followed 2 days later by another blister on the side of her left hand. Initially, both papules were round and white with erythematous edges (Appendix Figure 2, panel A), but they became dome-shaped, firm, smooth, umbilicated nodules 12–15 mm in size (Appendix Figure 2, panels B, C). A third lesion formed on the woman’s mid-upper back but was perforated through chafing from clothing. About 3 days after the appearance of the first lesion, the woman reported feeling unwell, fatigued, and feverish and had severe headaches. No lymphadenopathy was recorded. The lesions were persistently painful and hypersensitive, but none were cystic or became pustular. Instead, they became ulcerated, open, and dry (Appendix Figure 2, panel D), and all 3 resolved over a period of 6 weeks, leaving slight discoloration. After discovering the third lesion, the woman sought medical attention. Differential diagnoses included allergies, cellulitis, erysipelas, pyoderma gangrenosum, or granulomas caused by foreign bodies or insect bites (e.g., mango fly bites).

**Figure 2:**
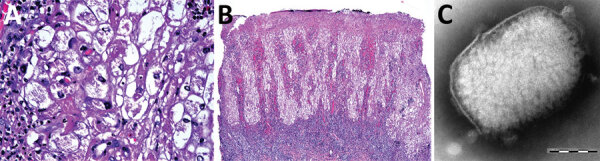
Diagnostic light and electron microscopy of tanapox lesion biopsies from a case-patient, South Africa, 2022. A) High-power photomicrograph of initial skin biopsy, showing prominent vacuolation of epidermal keratinocytes, granular intracytoplasmic inclusions, and intranuclear pseudoinclusions. Hematoxylin and eosin stain; original magnification ×400. B) Low-power photomicrograph of initial skin biopsy, showing a superficially eroded hyperplastic epidermis, with cytoplasmic pallor and a dense underlying superficial dermal lymphoid infiltrate. Hematoxylin and eosin stain; original magnification ×40. C) Negatively stained tanapox virus virion with surface tubules evident beneath the remains of the surrounding membrane. Virion dimensions were 159–327 nm × 186–289 nm. Scale bar indicates 100 nm.

Histopathologic examination of a lesion biopsy indicated a possible pox infection, given the presence of acidophilic intracytoplasmic inclusion bodies and cellular vacuolation (Figure 2, panel A). The lesion was confined to the epithelial layers with many cells that had vacuolated nuclei, and we observed ballooning cells in the deeper epithelial layer (Figure 2, panel B). Subsequently, we took an additional biopsy for electron microscopy and 2 lesion swab specimens for molecular characterization. After routine processing (Appendix), we observed numerous brick-shaped virions 287 nm x 221 nm with distinct surface tubules and generally an outer membrane layer (capsular form) (Figure 2, panel C). We used 1 dry swab sample for PCR analysis (Appendix), and partial sequence analysis indicated clustering with available TANV sequences (Appendix Figure 3). Attempts at full genomic sequencing and virus isolation (from the second swab specimen) were unsuccessful, possibly because of the limited clinical material available.

Recent surveys of the mosquito distribution in southern Africa have found that 2 culicine genera (*Culex* and *Mansonia*) comprise 91% of the mosquito population in the town of Skukuza (*11*). Weather conditions at the time of the case exposure were conducive to vector replication; recent rainfall was up to 147% higher than the average annual cumulative total (*12*), and ambient temperatures were 100°F–104°F (38°C–40°C) (*13*). In terms of virus reservoirs, a limitation of Monroe et al.’s model (*9*) was that the restricted range of recorded human cases determined the exclusion of many other primates with extensive geographic ranges. Our report extends this range beyond the most southerly predictions of the model, which increases the pool of potential reservoir hosts.

## Conclusions

The clinical findings for this reported case were in keeping with previous clinical reports of tanapox, and the diagnosis was supported by histopathology, electron microscopy, and molecular analyses. The importance and continuing relevance of histopathology and microscopy to the diagnosis and investigation of zoonotic disease were clearly illustrated. Given that tanapox is a vectorborne disease, many drivers, including anthropogenic destruction of wildlife habitats, environmental instability, and global climate change, may influence its emergence (*14*). Improved surveillance, including studies relating to the ecology and epidemiology of TANV in vectors, hosts, and humans, is warranted.

Appendix Additional information about tanapox, South Africa, 2022.
