# Supplementary material for: Tanapox, South Africa, 2022
Source: Emerg Infect Dis. 2023 Jun;29(6):1206–9. doi: 10.3201/eid2906.230326 (PMC10202874; doi:10.3201/eid2906.230326)
Supplement: Appendix — Additional information about tanapox, South Africa, 2022. [file 23-0326-Techapp-s1.pdf]

Article DOI: https://doi.org/10.3201/eid2906.230326

Tanapox, South Africa, 2022

Appendix

Methods

The first biopsy was routinely processed for light microscopy, and sections were stained with hematoxylin and eosin (*1*). The second biopsy was macerated in distilled water, and the supernatant was used to make grids for negative staining and transmission electron microscopy. Briefly, Formvar-coated, 300 mesh copper grids were placed on drops of supernatant for ten to 30 minutes, stained for five seconds either with 2% buffered phosphotungstic acid (pH 6.8) or with freshly prepared, 4% aqueous uranyl acetate, and viewed on an FEI Tecnai 12 BioTwin Spirit transmission electron microscope (https://www.thermofisher.com) with an Olympus Quemesa CCD camera and calibrated software (https://www.olympus-sis.com).

For PCR analysis, a dry swab was immersed in 300 µl sterile phosphate buffered saline and vortexed briefly. A volume of 140 µl of the supernatant was collected and nucleic acid extracted using the QiaAmp Viral RNA Minikit (Qiagen) in accordance with the manufacturer's protocol (https://www.qiagen.com). PCR amplicons of a 220 bp region of the insulin metalloproteinase-like protein (*2*) and a 1082bp region of the DNA polymerase gene (*3*) were sequenced (GenBank accession nos. OQ267780 and OQ230282, respectively), and phylogenetic analysis performed using MEGA X.

References

1. Suvarna SK, Layton C, Bancroft JD, editors. Bancroft's theory and practice of histological techniques. 8th edition. New York: Elsevier; 2019. https://doi.org/10.1016/B978-0-7020-6864-5.12001-8

2. Li Y, Meyer H, Zhao H, Damon IKGC. GC content-based pan-pox universal PCR assays for poxvirus detection. J Clin Microbiol. 2010;48:268–76. PubMed https://doi.org/10.1128/JCM.01697-09

3. Brettschneider H, Voster JH, Lane EP, van Wilpe E, Biden P, Dalton DL, et al. Molecular detection of Yaba monkey tumour virus from a vervet monkey. J S Afr Vet Assoc. 2013;84:1–5. https://doi.org/10.4102/jsava.v84i1.978

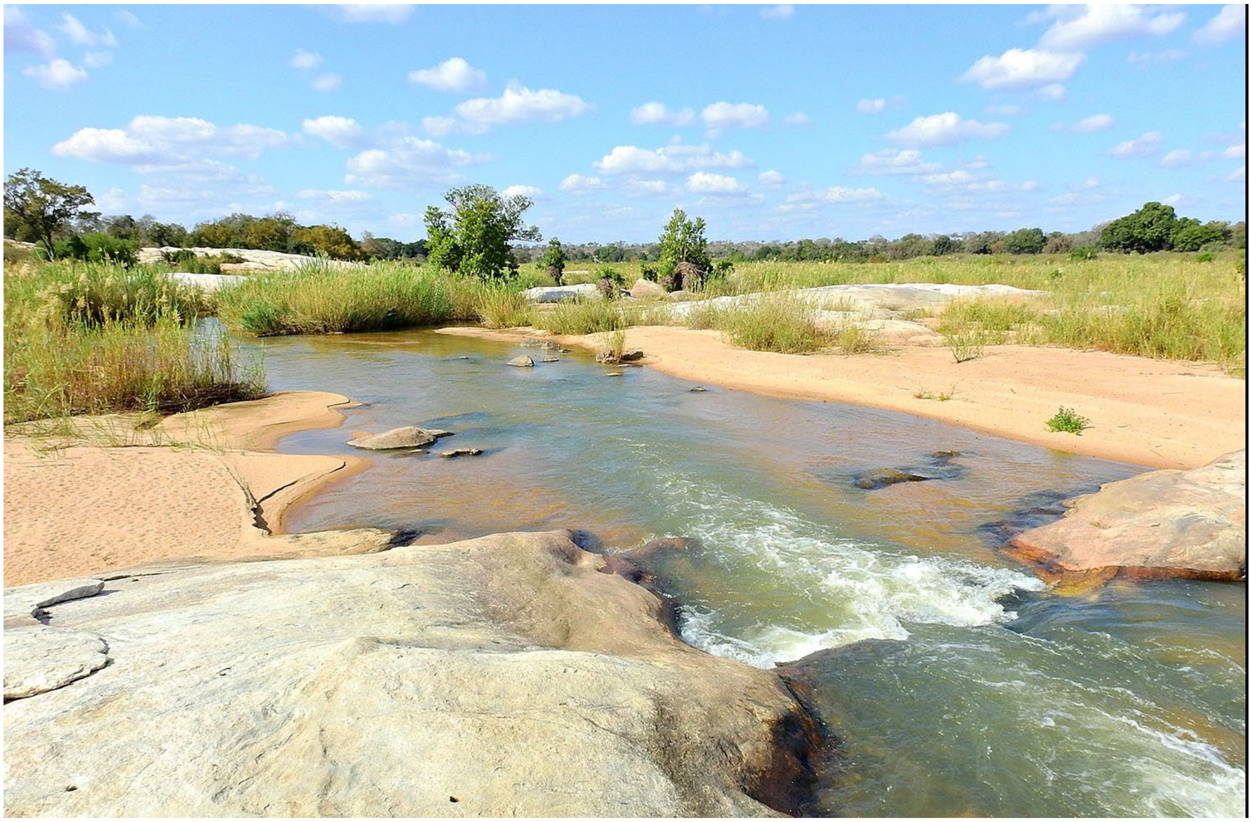

Appendix Figure 1. Banks of the Sand River, Kruger National Park, South Africa.

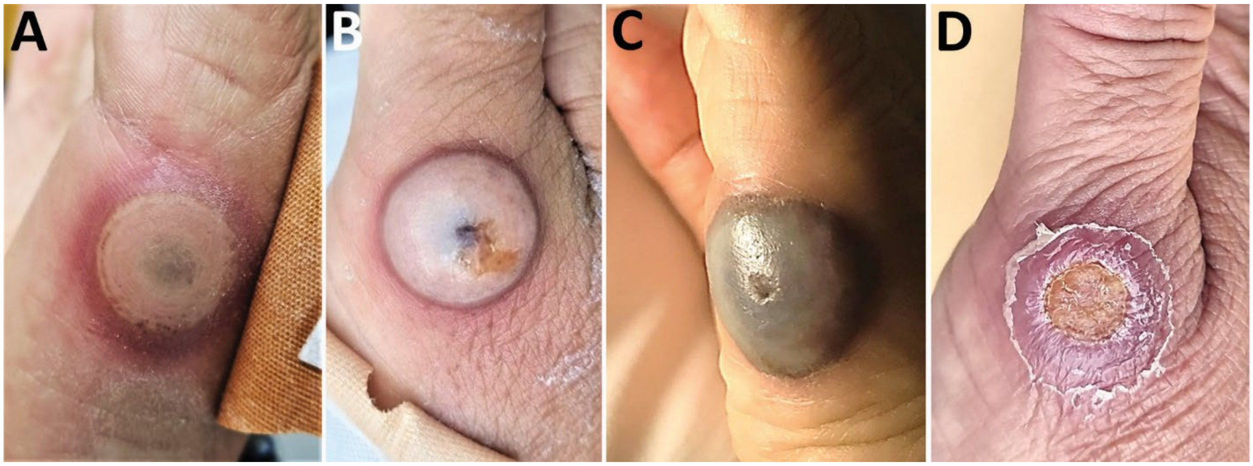

Appendix Figure 2. Development and morphology of tanapox lesions. A) Erythematous lesion with central discoloration, on the side of the left hand (12 days). B) Raised, nodular, umbilicated lesion on the base of the right-hand thumb (14 days). C) Umbilicated, violaceous lesion on the left hand (20 days). D) Dry, ulcerated lesion on the right thumb (27 days).

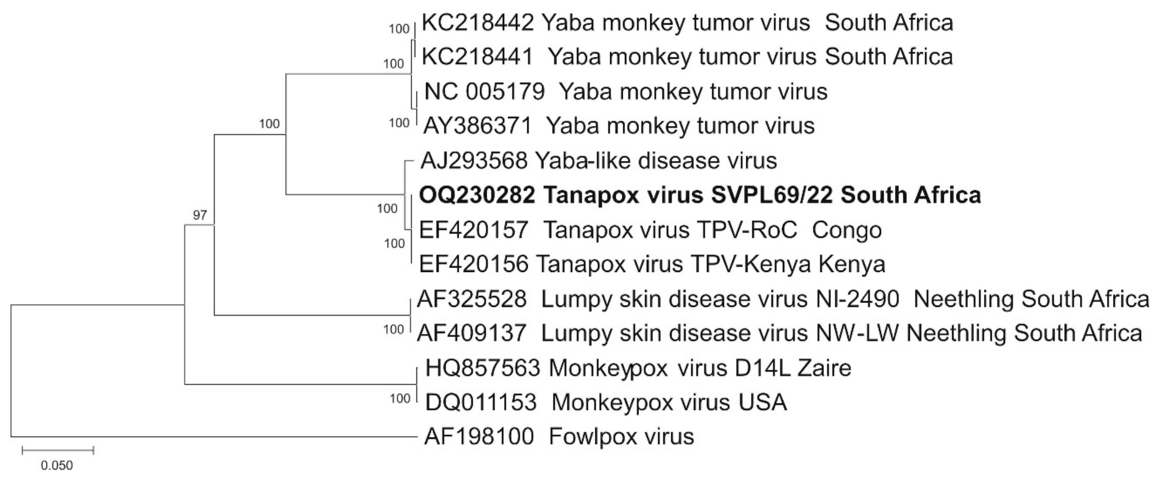

Appendix Figure 3. Neighbor-joining phylogenetic reconstruction using MEGA X of a partial 894bp region of the DNA polymerase gene showing the relatedness of selected pox viruses. Node values indicate bootstrap confidence values generated from 1000 replicates. Scale bar indicates substitutions per site. Sequence from this study is indicated in boldface.

.
